# The Reliability and Sensitivity of the National Institutes of Health Stroke Scale for Spontaneous Intracerebral Hemorrhage in an Uncontrolled Setting

**DOI:** 10.1371/journal.pone.0084702

**Published:** 2013-12-19

**Authors:** Adrian V. Specogna, Scott B. Patten, Tanvir C. Turin, Michael D. Hill

**Affiliations:** 1 Department of Community Health Sciences, Faculty of Medicine, University of Calgary, Calgary, Alberta, Canada; 2 Department of Clinical Neurosciences, Foothills Hospital, Calgary, Alberta, Canada; University of Münster, Germany

## Abstract

**Background and Purpose:**

The National Institutes of Health Stroke Scale (NIHSS) is commonly used to measure neurologic function and guide treatment after spontaneous intracerebral hemorrhage (ICH) in routine stroke clinics. We evaluated its reliability and sensitivity to detect change with consecutive and unique rater combinations in a real-world setting.

**Methods:**

Conservative measures of interrater reliability (unweighted Kappa (κ), Intraclass Correlation Coefficient (ICC_1,1_) and sensitivity to detect change (Minimal Detectable Difference (MDD)) were estimated. Sixty-one repeated ratings were completed within 1 week after ICH by physicians and nurses with no investigator intervention.

**Results:**

Reliability (consistency) of the NIHSS total score was good for both physicians vs. nurses and nurses vs. nurses (ICC=0.78, 95%CI: 0.58-0.89 and ICC=0.75, 95%CI: 0.55-0.87 respectively) in this scenario. Reliability (agreement) of items 1C and 9 were excellent (κ>=0.61) for both rater comparisons, however, reliability was poor to fair on most remaining items (κ:0.01-0.60), with item 11 being completely unreliable in this scenario (κ<0.01). The MDD_95_ of the total NIHSS score was ±10 and ±11 points for physician vs. nurse and nurse vs. nurse comparisons.

**Conclusions:**

The reliability of the NIHSS is good overall for ICH even in an uncontrolled setting. However, on repeated measurements changes in total NIHSS score of at least >=10 points need to be observed for clinicians to be confident that real changes had occurred within 1 week after ICH.

## Introduction

 The National Institutes of Health Stroke Scale (NIHSS) is a well known scale, originally designed to assess stroke severity in controlled clinical studies of ischemic stroke[1]. Despite this, it is now commonly used to measure neurologic function and guide treatment after spontaneous intracerebral hemorrhage (ICH) in day-to-day clinical settings as well[2]. Currently however, the sensitivity of the NIHSS for detecting changes after treatment is unclear, and reliability estimates from previous studies using distinct, controlled raters are over-estimated for routine settings where raters are often transient and interchangeable. Without knowing the reliability or sensitivity to detect change in uncontrolled settings with typical raters, it would be impossible to appropriately quantify clinically meaningful neurologic changes after treatment using this scale[3]. We evaluated the reliability and sensitivity to detect change of the NIHSS for ICH patients in a typical, routine clinical setting with a realistic set of consecutive raters.

## Methods

 The study protocol was approved by the University of Calgary Conjoint Health Research Ethics Board. We obtained a waiver of written consent for patients to conduct this study. A consecutive series of 48 patients with ICH were followed prospectively in a stroke unit at a university hospital. Patients were included if they were adults (>=18 years) and had an imaging-confirmed ICH. Patients were excluded only if they had an illness that interfered with neurological assessments, or paired-measurements were taken greater than four hours apart. 

 Raters of the NIHSS were physicians and nurses trained in stroke who were blinded to the study protocol. There was no specific, defined set of raters chosen for this study. Rather, raters were enrolled consecutively into the study and represented typical raters who would normally evaluate patients in routine settings but were not excluded based on their level of professional training or experience. Two raters completed NIHSS measurements within the first week after ICH. No formal training was provided for this study although it is a policy at our centre to ensure that all clinicians are NIHSS-certified prior to assessing stroke patients. 

 Interrater reliability of the total NIHSS score was quantified using an Intraclass Correlation Coefficient (ICC) model (1,1)[4]. This model was appropriate since all ratings were performed by a different set of raters[4]; which would be expected in routine settings since clinical rotations are often highly variable. Thus an interrater ICC (1,1) can be considered a realistic estimate of reliability for this scenario in contrast to a model 2 ICC which is used in the majority of reliability studies when a specific group of raters is defined *a prori*[5]. Interrater reliability of individual item scores was quantified using a conservative unweighted Kappa coefficient.

 Sensitivity to detect change of the total NIHSS score was estimated at different levels of confidence using the Minimal Detectable Difference (MDD)[5,6]. The MDD is a statistical measure that accounts for normal variability in clinician measurements over a large group of patients and identifies the smallest amount of change that is required to detect any improvement or decline in the natural units of a scale[5] while accounting for this normal variability. The MDD does not describe clinically meaningful changes in scores, rather it quantifies a level of statistical uncertainty surrounding specific NIHSS scores so clinicians can assess how likely they have captured 'true' improvement or worsening. Factors associated with absolute disagreement on individual scale items and magnitude of disagreement on the total NIHSS score between raters were investigated using logistic and linear regression respectively. The required sample size for this study was estimated to be at least 22 paired-ratings per rater comparison[7]. 

## Results

 Sixty-one pairs of ratings were completed across 38 patients. Ten patients were excluded because repeated measurements were taken greater than four hours apart. All 61 pairs of ratings were performed by 61 independent and unique combinations of physician and nurse raters. The characteristics of the patients included in each rater comparison are described in Table 1. Reliability of the NIHSS total score was good for both physicians vs. nurses and nurses vs. nurses in this scenario. The full results of reliability and sensitivity to detect change analyses are presented in Table 2. 

**Table 1:** Patient characteristics.

**Characteristic**	**Physician vs. Nurse Assessments (n=29 Pairs)**	**Nurse vs. Nurse Assessments (n=32 Pairs)**
Age (Years)	73 ± 9 (58 - 88)	68 ± 16 (38 - 88)
Sex (%Males)	66	56
GCS at Admission	13 (11 - 15)	14 (11 - 15)
Pre-Stroke mRS	0 (0 - 2)	0 (0 - 2)
Hemorrhage Location (%)		
Right	41	34
Left	59	59
Midline	0	6
Brainstem	0	3
Cerebellum	0	13
Lobar	41	22
Intraventricular	0	16
Putamen/Caudate	21	25
Thalamic	38	22

Data for age are presented as mean ± standard deviation (min-max). GCS is Glasgow Coma Score and is presented as median (min-max). mRS is Modified Rankin Score and is presented as median (min-max).

**Table 2:** The reliability (Kappa for items 1-11 and ICC for total score) and sensitivity to detect change (MDD) of the NIHSS in an uncontrolled clinical setting.

**Item**	**Physicians vs. Nurses**	**Nurses vs. Nurses**
1a. Level of Consciousness	0.26 (95% CI:0 - 0.54)*	0.54 (95% CI:0.26 - 0.83)
1b. LOC Questions	0.54 (95% CI:0.28 - 0.79)	0.32 (95% CI:0.04 - 0.59)
1c. LOC Commands	0.74 (95% CI:0.47 - 1.00)	0.65 (95% CI:0.33 - 0.98)
2. Best Gaze	0.08 (95% CI:0 - 0.36)*	0.45 (95% CI:0.18 - 0.72)
3. Visual	0.43 (95% CI:0.24 - 0.63)	0.39 (95% CI:0.12 - 0.65)
4. Facial Palsy	0.24 (95% CI:0.02 - 0.46)	0.52 (95% CI:0.22 - 0.83)
5a. Motor Arm: Left Arm	0.53 (95% CI:0.30 - 0.76)	0.80 (95% CI:0.58 - 1.00)
5b. Motor Arm: Right Arm	0.39 (95% CI:0.16 - 0.62)	0.62 (95% CI:0.40 - 0.84)
6a. Motor Leg: Left Leg	0.29 (95% CI:0.07 - 0.50)	0.72 (95% CI:0.51 - 0.93)
6b. Motor Leg: Right Leg	0.41 (95% CI:0.16 - 0.65)	0.67 (95% CI:0.43 - 0.91)
7. Limb Ataxia	0*	0.34 (95% CI:0.08 - 0.60)
8. Sensory	0.17 (95% CI:0 - 0.43)*	0.35 (95% CI:0.06 - 0.64)
9. Best Language	0.68 (95% CI:0.40 - 0.96)	0.78 (95% CI:0.50 - 1.00)
10. Dysarthria	0.35 (95% CI:0.06 - 0.64)	0.43 (95% CI:0.17 - 0.70)
11. Extinction and Inattention	0.17 (95% CI:0 - 0.46)*	0.26 (95% CI:0 - 0.55)*
Total Score	0.78 (95% CI:0.58 - 0.89)	0.75 (95% CI:0.55 - 0.87)
MDD_95_ of Total Score	± 9.64 Points	± 10.73 Points
MDD_80_ of Total Score	± 6.31 Points	± 7.02 Points
MDD_70_ of Total Score	± 5.10 Points	± 5.67 Points
MDD_60_ of Total Score	± 4.14 Points	± 4.61 Points
MDD_50_ of Total Score	± 3.32 Points	± 3.69 Points
MDD_40_ of Total Score	± 2.58 Points	± 2.87 Points
MDD_30_ of Total Score	± 1.90 Points	± 2.11 Points
MDD_20_ of Total Score	± 1.25 Points	± 1.39 Points
MDD_10_ of Total Score	± 0.62 Points	± 0.69 Points

^*^ Unreliable

MDD subscript is level of confidence (%). Reliability coefficients equal to zero indicate 'unreliable'.

 Rater disagreement (yes vs. no) using all paired-ratings (n=61) on item 1a was significantly associated with patient sex (OR for males: 9.73, 95% CI: 1.17-81.27), and lobar location (OR: 4.32, 95% CI: 1.14-16.33). Rater disagreement on item 5 was significantly associated with patient sex (OR: 4.26, 95% CI: 1.06-17.13) and patient age (OR per year older: 1.10, 95% CI: 1.02-1.17). Rater disagreement on item 6 was also significantly associated with patient sex (OR: 4.74, 95% CI: 1.34-16.74). For item 11, right-sided ICH was significantly associated with rater disagreement compared to the left or midline ICH (OR: 3.22, 95% CI: 1.04-9.93). Also, ICH located in the putamen or caudate was associated with significantly higher odds of disagreement amongst raters compared to all other locations (OR: 3.89, 95% CI: 1.11-13.65) for item 11. None of the aforementioned characteristics were associated with the magnitude of disagreement on the total score.

## Discussion

 Neurologic outcome scales such as the NIHSS are commonly used to assess neurologic function and determine how patients with stroke respond to treatment in day-to-day clinical settings. To our knowledge, this was the first study to evaluate the reliability and sensitivity to detect change of the NIHSS for ICH specifically and the first study to examine these properties for the NIHSS using a heterogeneous group of consecutive raters in an uncontrolled setting. Assessing the reliability of the NIHSS in an uncontrolled environment establishes a benchmark of what would be expected in daily practice, in the naturalistic setting of a tertiary care stroke program, and therefore the ICC estimated for the total NIHSS score in this study could be viewed as a conservative estimate of reliability[5]. We are confident that estimates presented in this study are generalizable to other routine stroke clinics but stress that they are not generalizable to settings where control of raters is implied such as in a randomized clinical trial of therapy.

 This study suggests that the reliability of the total NIHSS score was good in an uncontrolled setting but, as expected, it was lower than previous investigations with pre-defined raters[8], and may be affected by patient age, sex, and ICH location[9]. NIHSS measurements are never error-free in any scenario. The MDD is a statistical measure which explains this error and quantifies the smallest amount of change the NIHSS can accurately measure[5]. This study demonstrates that in an uncontrolled clinical setting, observed changes in the total NIHSS score (worsening/improvement) of 3 points, although may be considered *clinically meaningful* for some individual patients, over a large group of patients, can only be considered *real* with 50% certainty at best, due to natural errors in measurement, and the degree of error that affects individual NIHSS measurements is fairly substantial, despite good observed reliability overall.

 Clinicians should define *clinical improvement* outside the range of the natural statistical error of NIHSS scores, specifically it must be defined as >=10 points (if nurses and physicians are making the measurements) further from the baseline/previous score, to conclude that observed measurements reflect *real* neurologic changes, with any substantial certainty (95%). 

 As with many previous studies of reliability we assessed consistency and agreement between raters while taking multiple measurements within the same set of patients. Reliability studies attempt to quantify and describe the interaction between raters and patients in different scenarios, thus the unit of analysis in reliability studies is ‘ratings’ versus ‘patients’ which is atypical for most clinical studies. Specifically, reliability coefficients are measures which describe rater-patient interactions, and therefore can only be valid if the combination of raters, patients, and times of assessment are independent and mutually exclusive across each pair of ratings, as they were in our study. Further, it is reiterated that this study did not assess clinically meaningful changes on the NIHSS. Rather, this study evaluated the errors associated with rating the NIHSS using a statistical distribution-based method. Clearly, further studies are still needed to identify what magnitude of change is necessary on the NIHSS to observe clinically important changes. Assumptions cannot be made regarding clinically important changes on a scale if it is unknown what strength of signal is required to overcome the natural error of a scale and register a change to begin with. Thus, this study provides evidence for these future investigations. 

## Conclusion

 The NIHSS total score is reliable for ICH even in an uncontrolled setting, however, good reliability does not imply good sensitivity for detecting true neurologic function. Thus, clinicians need to be aware of important patient characteristics that may be associated with increased variability among repeated measurements.
